# Thermosensitive Hydrogel Wound Dressing Loaded with Bacteriophage Lysin LysP53

**DOI:** 10.3390/v14091956

**Published:** 2022-09-03

**Authors:** Changchang Li, Raphael Nyaruaba, Xiaowei Zhao, Heng Xue, Yuhong Li, Hang Yang, Hongping Wei

**Affiliations:** 1 CAS Key Laboratory of Special Pathogens and Biosafety, Center for Biosafety Mega-Science, Wuhan Institute of Virology, Chinese Academy of Sciences, Wuhan 430071, China; 2 College of Life Sciences, University of Chinese Academy of Sciences, Beijing 100049, China; 3 The State Key Laboratory Breeding Base of Basic Science of Stomatology (Hubei-MOST) & Key Laboratory of Oral Biomedicine, Ministry of Education, School of Stomatology, Wuhan University, Wuhan 430079, China

**Keywords:** bacteriophage, lysin, LysP53, hydrogel, poloxamer 407, wound infection, drug resistance, *Acinetobacter baumannii*

## Abstract

Wound infections are prone to attacks from infectious pathogens, including multidrug resistant bacteria that render conventional antimicrobials ineffective. Recently, lysins have been proposed as alternatives to conventional antimicrobials to tackle the menace of multidrug resistance pathogens. The coupling of lysins with a material that will cover the wound may prove beneficial in both protecting and treating wound infections. Hence, in this study, a Gram-negative lysin, LysP53, was coupled with a thermosensitive hydrogel, poloxamer P407, and its efficacy to treat wound infection was tested. In vitro, the addition of LysP53 to the poloxamer did not affect its thermosensitive characteristics, nor did it affect the hydrogel structure. Moreover, the lysin hydrogel could hydrolyze the peptidoglycan, demonstrating that it may have bactericidal activity. Up to 10.4% of LysP53 was released from the hydrogel gradually within 24 h, which led to a 4-log reduction of stationary phase *Acinetobacter baumannii*. Lastly, the lysin hydrogel was found safe with no cytotoxic effects observed in cells. Ex vivo, LysP53 hydrogel could inhibit bacterial growth on a pig skin decolonization model, with 3-log differences compared to non-treated groups. Overall, our results suggest that lysin-loaded hydrogels may provide a novel solution to treat wound infections caused by resistant bacteria.

## 1. Introduction

The skin is an important and effective barrier against the environment. It plays a vital role in preventing harmful microorganisms and foreign factors, such as exogenous substances and dehydration [1]. The skin is the largest and outermost organ covering the whole body. Skin and soft tissue infections (SSTIs) are the most common type of infection worldwide, accounting for about 7% to 10% of hospitalized patients [2] In the United States alone, over 14 million people are affected by SSTIs yearly [3,4]. Trauma occurs when a tissue is destroyed or its cellular integrity is impaired due to mechanical, physical, or metabolic related problems [5]. Many people today suffer from open wounds of the skin arising from cuts, burns, diseases (such as diabetes), or surgical operations [6]. Depending on the duration and nature of the healing process, skin trauma can be classified as acute or chronic. Bacterial colonization usually occurs in chronic wounds and is considered to be the main cause of chronic inflammation [7]. Traditionally, antibacterial agents have been used to prevent and treat local wound infections. However, this treatment has been impeded by the emergence of multidrug resistant (MDR) bacteria that are capable of colonizing wound infections and exacerbating the medical condition of affected individuals [8].

*Acinetobacter baumannii*, a Gram-negative opportunistic bacterium, can cause severe wound, skin, and soft tissue infection [9,10,11,12]. In 2017, the World Health Organization (WHO) classified *A. baumannii* to be among the ESKAPE pathogens, an acronym comprising of six highly virulent and drug resistant pathogens (*Enterococcus faecium*, *Staphylococcus aureus*, *Klebsiella pneumoniae*, *Acinetobacter baumannii*, *Pseudomonas aeruginosa*, and *Enterobacter* spp.) [13]. Due to this, it has been proposed that the discovery, design, and development of new and alternative antimicrobial therapies against such pathogens is crucial [14]. Since their discovery, bacteriophages (phages) have been explored as a suitable alternative to antibiotics. Bacteriophage lysins are proteins generated by phages to release their progeny from bacterial host cells [15]. Since phage lysins do not have signal sequences, they usually depend on holin protein to form pores in the inner membrane of infected cells, which allows them to access the peptidoglycan, causing rapid cell lysis [16]. However, it has been found that lysins can be recombined as enzymes and applied exogenously to cause rapid cell lysis due to the absence of a membrane to inhibit their access to the bacterial cell wall [17]. Because of their ability to rapidly lyse pathogenic bacterial cells upon contact with the peptidoglycan (lysis from without), phage-derived lysins are being explored as a promising new class of antimicrobials to combat antimicrobial resistance (AMR) arising from MDR pathogens. Unlike bacteriophages to which bacteria can develop resistance easily [18,19,20], no resistance was found after 40 days of incubation of bacteria with the phage-derived lysin at sub-minimum inhibition concentration [21]. Several factors also render lysins as suitable alternatives to antibiotics, including rapid bactericidal activity and the inability to interfere with the normal flora [22] due to better selectivity. In our previous study [23], we found one lysin (LysP53) that showed good antimicrobial activity against *A. baumannii* and other pathogens, such as *E. coli*, etc. Due to its nature of protein, the stability and form of LysP53 when applied topically may be important for its application in wound infections.

Hydrogels are defined as a special class of three-dimensional cross-linked polymer structures with excellent ability to expand and retain large amounts of water or biological fluids without disrupting their network [24]. Due to their structural similarity to natural extracellular matrix, good biocompatibility and biodegradability, hydrogels have attracted great attention in biomedical applications, such as drug delivery, medical implants, and tissue engineering [25]. Despite the increasing threat of antibiotic resistant strains to colonized wounds, hydrogel dressing containing antimicrobial agents is still being used as the first choice of therapy [26]. Therefore, a suitable replacement to antibiotics can be coupled with hydrogels to reduce chances of wound infections arising from MDR pathogens. To tackle this, in this study, the lysin LysP53 was used as a replacement for conventional antibiotics and loaded into a thermosensitive hydrogel made of Poloxamer 407. Poloxamer 407 is a hydrophilic, non-ionic triblock copolymer composed of a hydrophobic residue of polyoxypropylene (POP) flanked by two hydrophilic units of polyoxyethylene (POE) [27]. One of the main factors that makes this material suitable for topical application is its thermo-reversibility. Poloxamer 407 exists as a liquid at room temperature but can form a gel when applied at body temperature [27]. Since Poloxamer 407 is hydrophilic and non-ionic, it is hypothesized to stabilize phages by forming hydrogen bonds and providing steric hindrance to minimize phage aggregation [8]. Hence, Poloxamer 407 has been used to deliver whole phages to treat *A. baumannii* wound infections [8], *Enterococcus faecalis* root canal infection [28], and *Staphylococcus aureus* bone infection [29]. In these studies, the hydrogels formed showed varied phage activities against bacterial infections and it was also shown that the Poloxamer 407 could be used for the long-term storage of bacteriophages (at 4 ℃) without affecting their activity [8]. Despite the efficacies, no study has attempted to check the suitability of phage-derived lysins (which may be more suitable than phages, as described previously) to treat bacterial infections using the Poloxamer 407.

Therefore, for the first time, in this study, a Gram-negative lysin was used in combination with the Poloxamer 407 to treat wound infections. To achieve this objective, both in vivo and ex vivo experiments were performed to demonstrate its suitability in treating MDR *A. baumannii* over a long-time exposure on skin wounds.

## 2. Materials and Methods

### 2.1. Bacterial Strains

*E. coli* BL21(DE3) cells were cultured in lysogeny broth (LB) medium aerobically at 37 °C and used for cloning and protein expression. *A. baumannii* WHG40137 were cultured in LB medium aerobically at 37 °C and used for bactericidal activity detection.

### 2.2. Cloning, Expression, and Purification of LysP53

The open-reading frame (ORF) encoding the putative endolysin LysP53 (ORF37) was amplified from *A. baumannii* phage 53 (NCBI: MW590698) using the primers (5′-3′): 53P37-F:ctttaagaaggagatataccatggatgacgatgacaacaaaacgta and 53P37-R: tggtggtggtggtggtgctcgagccccgccaattcaaagtgtgggct and cloned into the *Nco*I and *Xho*I sites of the plasmid pET28a(+). Subsequently, plasmids were transformed into *E. coli* BL21(DE3) cells and further confirmed by sequencing.

*E. coli* BL21 (DE3) containing the pET28a-LysP53 was cultured to an OD_600_ of 0.5~0.6 in 500 mL LB medium containing 500 µg/mL kanamycin, then induced with 0.5 mM isopropyl β-D-thiogalactoside (Thermo Scientific, Waltham, MA, USA) at 16 °C for 16 h. Bacterial cells were then harvested by centrifugation at 7370× *g* for 10 min at 4 °C, washed once with 20 mM imidazole and then resuspended in 20 mM imidazole. Cells were lysed on ice by a cell disrupter and then centrifugated at 7370× *g* for 20 min at 4 °C. The resultant supernatant was filtered through a 0.22 µm syringe filter and subjected to a nickel nitrilotriacetic acid affinity column (GE Healthcare, Chicago, IL, USA) following the manufacturer’s instructions. Fragments collected after eluting with 250 mM imidazole were pooled, and dialyzed against 20 mM Tris-buffer (contain 0.5 mM NaCl, pH 6.5) overnight at 4 °C.

### 2.3. Preparation of a Lysin-Loaded Hydrogel

A commercial hydrogel Poloxamer 407 (Sigma-Aldrich, St. Louis, MO, USA) and lysin LysP53 were used to develop the LysP53 hydrogel. Firstly, the Poloxamer 407 powder was prepared into 20% solution using ultra-pure water, and then dissolved at 4 °C. LysP53 was lyophilized before use and dissolved in the poloxamer solution (~200 µg LysP53 in 0.4 mL poloxamer solution). This resulted in a colorless and transparent lysin-loaded poloxamer hydrogel solution (LysP53 hydrogel).

### 2.4. Morphology of Hydrogels

The morphology of hydrogels was determined by scanning electron microscopy (SEM). Briefly, the LysP53 hydrogel formulation was incubated at 37 °C for 10 min to form a rigid gel, followed by instant freezing with liquid nitrogen to maintain the structure of the formed hydrogel. The frozen hydrogel was freeze dried further for 45 h to remove all incorporated water. Finally, hydrogels were coated with gold (Hummer VI; Technic Inc., Anaheim, CA, USA) before observation under a SEM (SEM, SU8010, Chiyoda, Tokyo, Japan) at an accelerating voltage of 3 kv and 1000× magnification.

### 2.5. In Vitro Release of LysP53 from the LysP53 Hydrogel and Bactericidal Assay

A volume of 400 μL LysP53 hydrogel solution was pipetted into a microtube and incubated at 37 °C for 10 min to allow gel formation. An amount of 100 μL of Tris-buffer was added to the hydrogel and incubated further at 37 °C. Aliquots were taken at different time points of 1 h, 2 h, 3 h, 6 h, 12 h, and 24 h, respectively, for testing the concentration of LysP53 released into the solution as well as bactericidal activities. The concentration of LysP53 was determined using a Pierce BCA protein assay (Thermo Scientific) according to the manufacturer’s instruction. SDS-PAGE was run to determine the composition of the proteins in the aliquots collected at 24 h, 48 h, 72 h, and 96 h. For bactericidal activity test, *A. baumannii* cells grown to a stationary phase at 37 °C overnight were washed and resuspended in Tris-buffer. The bacterial suspensions were then mixed with 100 μL of LysP53 hydrogel solutions collected at different time points, and incubated for 60 min at 37 °C.

### 2.6. Peptidoglycan Hydrolysis Experiment

Agar powder was added to the overnight bacterial culture of *A. baumannii* for sterilization. The agar was then poured into a sterile Petri dish and solidified at room temperature. Small volumes of 10 μL each of different solutions containing either Tris-buffer, blank hydrogel, LysP53 or LysP53 hydrogel were dropped at different positions on the plate. The plate was finally incubated overnight at 37 °C.

### 2.7. Cytotoxicity Test

The LysP53 hydrogel was first incubated in equal volumes of Dulbecco’s modified Eagles medium (DMEM; Sigma, St. Louis, MO, USA) at 37 °C for 12 h and 24 h, and the whole medium containing LysP53 released from the hydrogel collected. To investigate the cytotoxicity of the LysP53 in the harvested medium, Caco-2 cell was used, with the same volume of free medium as a control. The cells were cultured in DMEM supplemented with 2% fetal bovine serum and 1% penicillin/streptomycin at 37 °C under a humidified atmosphere containing 5% CO_2_. Caco-2 cells were seeded into 96-well plates at a density of 2 × 10^4^ cells/well. After 24 h, the cell culture medium was exchanged with the harvested medium (100 µL). After 24 h incubation, the cell viability was measured by CCK-8 assay. The normal cell culture medium was used for a control group (cell viability, 100%).

### 2.8. Ex Vivo Studies

With slight modification, the ex-vivo experimental method was performed as previously described [8]. The pigskin used in this study was shaved and frozen at −20 °C. During the experiment, the remaining fat layer was removed from the frozen skin slices with a scalpel, and the skin was further cut into 1.4 × 1.4 cm small pieces. The small pieces were disinfected with 75% ethanol, washed with sterile ultrapure water, wiped dry with sterile (Autoclaved) paper towel, and put into a 12 well plate. The epidermis was removed leaving a dermal wound about 4–5 mm deep and 4 mm in diameter. The skin area (including the wound) was washed with sterile ultrapure water. The samples used for Firmness test were stained (for better visibility) and injected into the wound, then incubated at 37 °C to form a rigid gel. Then, the adhesion of LysP53 hydrogel was examined under tension (bending at the ends of the skin), bending, torsion, and compression. For the bacterial clearance of *A. baumannii* from infected pig skin wounds experiment, *A. baumannii* cultured overnight was diluted 1000 times with fresh LB medium and 50 μL of the bacterial culture was added to each wound area to allow bacteria to adhere to the skin. Then, 50 μL Tris-buffer, blank hydrogel, or LysP53 hydrogel were applied to infected wounds. After incubating the plate at 37 °C for 24 h, the bacteria were harvested by gently rubbing the wound and adding 500 µL Tris-buffer. Bacterial plate count method was used to evaluate the remaining bacteria in each group.

## 3. Results

### 3.1. Characteristics of Thermosensitive Hydrogels

**Figure 1:**
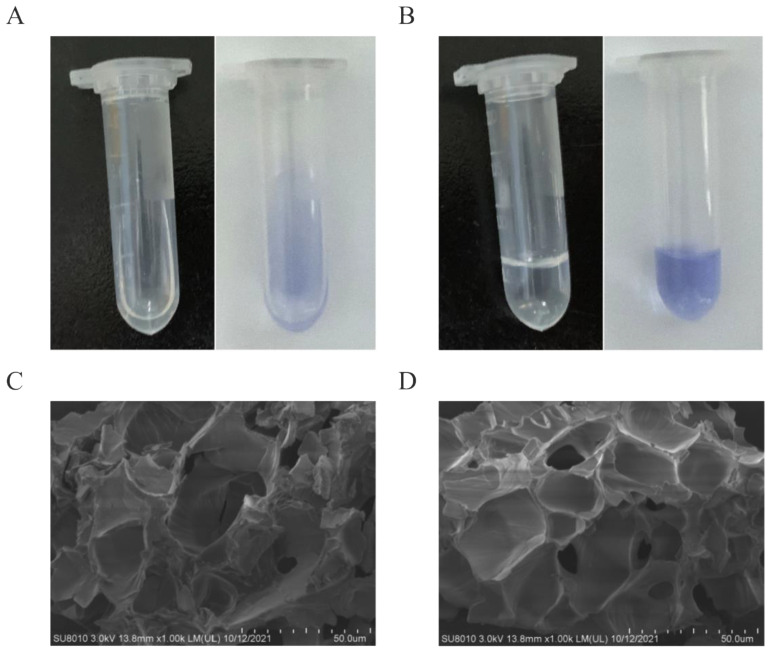
Images of the hydrogel solution and the LysP53 hydrogel solution containing crystal violet at (**A**) room temperature (flowable solution) and (**B**) 32 °C (rigid gel). The SEM micrographs of the Poloxamer 407 hydrogel (**C**) and LysP53-loaded Poloxamer 407 hydrogel (**D**). The addition of LysP53 does not affect the three-dimensional structure of the poloxamer 407 hydrogel. A and B are microtubes lying horizontally.

Due to its thermo-reversible properties, Poloxamer 407 exists in a fluidic state at room temperature. This facilitates administration and gel state above sol-gel transition temperature at body temperature promoting the prolonged release of pharmacological agents. As shown in Figure 1, the LysP53 hydrogel can flow freely at room temperature (~22 °C) (Figure 1A) and then form a solid gel at 32 °C (Figure 1B). This transition is important as the lysin can be applied at room temperature (free flowing) and gel formed at skin surface temperature (ranges between 32 °C to 35 °C) to allow for the slow releasing of lysin into wound site [30]. No difference in gelation temperature was observed between blank and lysin-loaded hydrogel solutions, indicating that the incorporation of lysin P53 had no effect on the gel network of Poloxamer 407. SEM was used to observe the structure of the thermosensitive hydrogel. Under SEM (Figure 1C), the hydrogel has a typical porous structure with a pore size of about 29.99 ± 8.38 µm quantified by ImageJ (n = 30). The pores are oval and crisscross, which is conducive for the free diffusion and flow of hydrophilic compounds or nanoparticles from the polymer network. Lysins, including LysP53 range in the nm scale, thus they can diffuse freely and be released to kill bacteria. When LysP53 was added to the Poloxamer 407 hydrogel, no effects on the formation of the three-dimensional structure of the gel was observed (Figure 1D). In addition, LysP54 has a pI (iso-electric point) of 6.57, which is near neutral pH hence has an overall net zero charge. This mean that it can freely interact with the Poloxamer 407 hydrogel.

### 3.2. LysP53 Hydrogel Can Hydrolyze Peptidoglycan and form a Hydrolytic Circle

**Figure 2:**
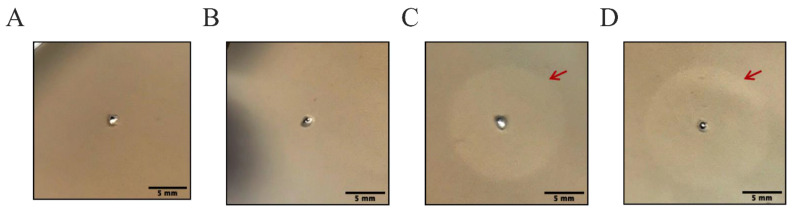
Testing hydrolysis activities of Tris-buffer (**A**), blank hydrogel (**B**), LysP53 (**C**), and LysP53 hydrogel (**D**). LysP53 and LysP53 hydrogel showed hydrolytic activity, while the Tris-buffer and blank hydrogel had no activity. Scale bar of 5 mm.

The peptidoglycan hydrolysis experiment was used to detect the hydrolysis activity of LysP53 to peptidoglycan. Different from the in vitro time-killing test under dynamic conditions, the hydrolysis experiment can evaluate the inhibitory effect of hydrogels in a static environment. The peptidoglycan hydrolytic circles of Tris-buffer, blank hydrogel, LysP53, and LysP53 hydrogel are shown in Figure 2. It can be seen that the buffer and the blank hydrogel did not show the hydrolytic circles (Figure 2A,B), while the diameters of the hydrolytic circles of fresh LysP53 (Figure 2C) and the LysP53 hydrogel (Figure 2D) were about the same (15.39 ± 0.09 mm and 15.54 ± 0.013 mm, respectively). This indicated that the lysin contained in the hydrogel could be released to perform hydrolysis effectively even in a static environment.

### 3.3. Release Rate and Bactericidal Activity of LysP53 Hydrogel at 37 °C

**Figure 3:**
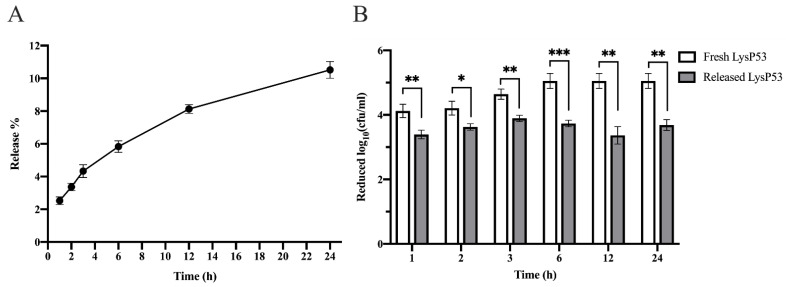
Analysis of LysP53 release rate, activity, and stability after release from LysP53 hydrogels at different times. (**A**) The cumulative release rates of LysP53 from the LysP55 hydrogel at different times. (**B**) Comparison of the activity of LysP53 hydrogel and freshly prepared LysP53. Freshly prepared LysP53 had a higher reduction rate, while the LysP53 hydrogel retained a steady reduction rate of ~3 logs (* <0.05, ** <0.01, and *** <0.001).

The cumulative release rate of LysP53 from the hydrogel was about 10.5% after 24 h (Figure 3A). During the time period, the released lysin from the hydrogel maintained a mean log reduction of 3.62 (±0.23) bacterial biomass at different time points (Figure 3B). However, this number was significantly lower compared to that of the freshly prepared lysin (4.69 (±0.45)) at a similar concentration. This was probably because the freshly prepare lysin could wholly interact with the bacteria while the LysP53 released from the hydrogel gradually, and thus could not fully access the bacteria. Despite this, a log reduction of 3.62 (±0.23) was still found to be good enough.

### 3.4. Cytotoxicity of LysP53 Hydrogel

**Figure 4:**
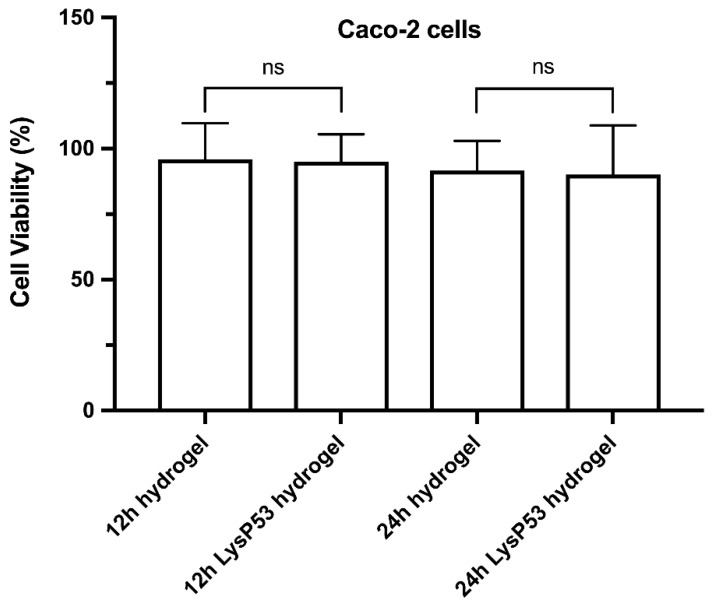
Relative viability of Caco-2 cells exposed to different release times of hydrogel only and LysP53-loaded hydrogel. A >90% cell viability was maintained at all conditions and times. Ns—not significant.

Caco-2 cells were used to evaluate the safety of LysP53-loaded hydrogel. Considering the fact that hydrogels are only active once they can diffuse or infiltrate, hydrogel only and LysP53-loaded hydrogels were added at different times in a cell plate containing Caco-2 cells to observe their effects on the growth rate of the cells as seen in Figure 4. After 24 h, the CCK-8 reagent was added to the cells containing hydrogels for measurement. CCK-8 analysis showed that the Caco-2 cells maintained good cell viability. Whether Caco-2 cells were exposed to 12 h or 24 h solutions released from the hydrogel only and LysP53 hydrogel, the relative viability of cells was more than 90%.

### 3.5. Ex Vivo Model

**Figure 5:**
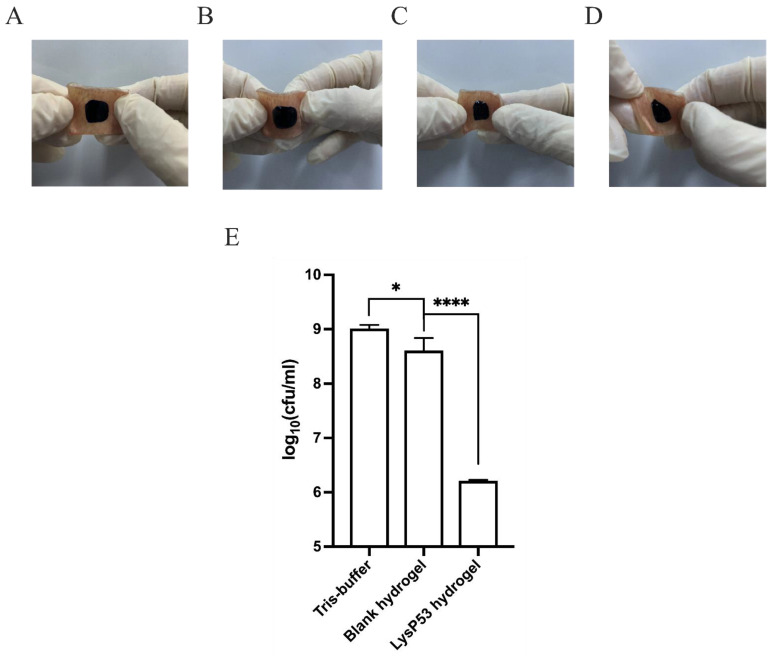
Photographs of the stretching (**A**), bending (**B**), compressing (**C**), and twisting (**D**) processes of LysP53 hydrogel. (**E**) *A. baumannii* concentrations were lower in the pig skin infection model treated with LysP53 hydrogel than those treated with Tris-buffer and blank hydrogel for 24 h (* <0.05, **** <0.0001).

The antibacterial effects of LysP53 hydrogel on wound infection were studied ex vivo using a pig skin model. At 37 °C, the hydrogel solution became rigid and formed a gel at the wound site. Even under the pressure of stretching, bending, torsion, and compression, the hydrogel still firmly attached to the wound (Figure 5A–D). As shown in Figure 5E, the LysP53 hydrogel could significantly prevent the growth of *A. baumannii* during the 24 h treatment period compared to the Tris-buffer and the blank hydrogel treatment group. Compared to the mean 9.01-log value in the Tris-buffer group and 8.6-log value in the blank hydrogel, there was only about 6.2-log numbers of the bacteria in the LysP53 hydrogel group. The mean 0.41-log difference between the Tris-buffer group and blank hydrogel group was thought to arise from the firm attachment of the blank hydrogel to the pig skin. This attachment may slightly inhibit some portion of the bacteria (especially those in direct contact with the hydrogel) from growing as compared to Tris-buffer, which does not form a gel on the skin.

## 4. Discussion

The increase of skin and soft tissue infections (SSTI) caused by multidrug resistant (MDR) pathogens is a global concern. Existing treatment protocols and guidelines on how to treat these pathogens are scarce [31]. The first-line agents are polymyxins; however, colistin-resistant *A. baummanii* can be found in as high as 50% of Carbapenem resistant *A. baumannii* [32,33]. Therefore, alternatives to antibiotics are important for treating infections caused by MDR pathogens. Among these alternatives, lysins have been explored as a potential replacement to antibiotics. Unlike antibiotics, several factors have hampered the general application of phages and lysins in clinical settings. These factors have been reviewed elsewhere [16,34,35,36]. One of them is the lack of a legal framework that explicitly guides the usage of lysins and phages as medical products for humans [37]. Despite these hurdles, studies have shown that phages and lysins can indeed be used to treat patients successfully, although they were often considered as alternatives after drug resistance tests [38,39,40]. However, there is merit in exploring their potential in treating different types of infections, including wound infections. Hence, in this study, the lysin LysP53 was loaded into a thermosensitive hydrogel and used to treat wound infections. The LysP53 hydrogel was used due to its attractive advantages.

First, LysP53 has been shown to have good bactericidal activities against MDR *A. baumannii* and other Gram-negative bacteria [23]. Similarly, in this study, the in vitro released-LysP53 from the hydrogel had a good bactericidal activity of 3.61 (±0.23) logs (Figure 2 and Figure 3), indicating that the gelation process has little effect on LysP53.

Second, the thermosensitive property of Poloxamer 407 makes topical application of LysP53 hydrogel easy. When the ambient temperature is low, lysin hydrogel is in a free-flowing state and is easy to be used quantitatively. However, when the ambient temperature is greater than 32 °C, it will solidify rapidly and adhere to the wound.

Lastly, the lysin hydrogel is safe and can attach to wound sites under stretching, bending, twisting, or even compression. This not only covered the wound site, thus protecting the wound from environmental bacteria but also provided a long-lasting antibacterial environment to kill the bacteria in the wound. Neither 12 h nor 24 h solution released from LysP53 hydrogel after incubation showed significant cytotoxic effects on Caco-2 cells, a cell line used to study the mechanisms of drug absorption and transportation [41,42]. The *A. baumannii* pig skin infection model also showed that the lysin hydrogel was highly efficient and was able reduce the number of the bacteria at the wound site from 10^9^ CFU to 10^6^ CFU, which aided in the clearance of the bacteria from the wound site.

However, although bacteria reduced from 10^9^ CFU to 10^6^ CFU, the bacterial level at the wound site was still high. In future, this could be tackled by using different dosages, i.e., applying a new hydrogel to the wound site after 24 h or increasing the lysin concentration in the hydrogel before topical application at the wound site. In addition, other shortcomings arising from the study include: the long-term storage stability of LysP53 in the thermosensitive Poloxamer 407, which is critical for developing commercially viable phage product but was not assessed; and the study involved only one ex vivo animal model, cell line, pathogen, and lysin. In future, these limitations will be addressed, and clinical trials of the lysin hydrogel are needed to confirm its efficacy and safety in treating wound infections.

## 5. Conclusions

In summary, this study showed that a Gram-negative bacteria lysin LysP53 can be loaded into a Poloxamer 407. The formed hydrogel may be an ideal dressing for treating wound infections caused by MDR *A. baumannii.* The formation of lysin hydrogel did not affect the physical and structural state of the poloxamer but also maintained the bactericidal activity of LysP53. The lysin hydrogel can adhere to the wound, release lysin continuously, and exert bactericidal effects to aid clearing of the bacteria. This study paves way for the topical application of other related lysins to prevent wound infections.
